# Late-onset Pompe disease manifests in the brain

**DOI:** 10.1016/j.ymgmr.2019.100488

**Published:** 2019-06-21

**Authors:** Josef Finsterer

**Affiliations:** Krankenanstalt Rudolfstiftung, Messerli Institute, Postfach 20, 1180 Vienna, Austria

**Keywords:** Glycogenosis, Metabolic myopathy, Cerebrum, Vasculopathy, Myelination, White matter lesions

Letter to the Editor

With interest we read the article by Schneider et al. about 19 patients with late-onset Pompe disease(LOPD) who did not differ with regard to Fazekas scores(FSs) for assessment of periventricular and deep white-matter lesions(PVWMLs, DWMLs) from 28 age-, sex-, and cerebrovascular risk factor(CVRF) profile-matched controls [1]. We have the following concerns.

We do not agree with the conclusions that WMLs in LOPD result from concomitant CVRFs rather than from LOPD [1]. Among LOPD patients 1 had no CVRFs but a total FS of 1 [1]. Occurrence of WMLs in this patient should be explained. Furthermore, the article does not compare the quality of CVRF control in LOPD and controls. It is conceivable that hyperlipidemia (*n* = 14), arterial hypertension (*n* = 13), obesity (*n* = 4), diabetes (*n* = 2), smoking (n = 1), were better controlled in LOPD patients than in controls. Thus, we should know in patients and controls the drugs they were taking, blood pressure values, HbA1c values, and serum lipid levels. We should also be informed if the combination of risk factors differed between the two groups. If patients were taking fewer drugs for controlling their CVRFs it is possible that the influence of CVRFs on the development of WMLs was lower in LOPD than in controls. A further argument for WMLs as a feature of LOPD is that WMLs also develop in children under ERT who do not carry any CVRFs [2]. It is also conceivable that ERT attenuated the development of WMLs attributable to LOPD in LOPD patients. Since patients with LOPD may develop dilative arteriopathy [3] and since glycogen may be stored directly in the brain [4], it cannot be excluded that WMLs are in fact a manifestation of LOPD.

To ultimately answer the question if WMLs are a phenotypic features of LOPD or a consequence of the CVRF profile, patients and controls without any CVRF should be compared.

## Declaration of competing interests

There are no conflicts of interest.
